# Medical Opinion and Movement

**Published:** 1908-02-01

**Authors:** 


### Medical Opinion and Movement.

During recent times pancreatic lesions have attracted the attention both of the physician and of the surgeon, and the pathology of the affections of this organ are still so obscure that any investigation of the subject is to be welcomed. An interesting contribution to the literature is Dr. Heineke's essay on "Rupture of the Pancreas" in the current issue of the "Archiv. f. Klin. Chirurgie," in which the history of four cases of this rare lesion is fully given. The author distinguishes two classes of rupture: (1) those complicated with a lesion to some other abdominal viscus; (2) uncomplicated ruptures; the first class being the more frequent. The rarity of either can be judged from the fact that among 9,500 autopsies made during seven and a half years at the Pathological Institute at Leipzig only one example of the first division was noted. The author gives some interesting facts regarding isolated ruptures, of which 19 cases appear to have been recorded; in two of these the lesion was overlooked at the exploratory operation. On the whole the prognosis of the second lesion is favourable, provided the case is diagnosed early. The chief dangers appear to be hæmorrhage, and the necrosing action of extravasated pancreatic juice upon the omentum and peritoneum. The diagnosis is often a matter of great difficulty, and the author does not consider Cammidge's or Sahli's reaction of much help as diagnostic aids.

At a recent meeting of the Academy of Medicine in Paris Professor Chantemesse has raised a cry of alarm which undoubtedly calls for serious consideration. He predicts the possibility of a cholera epidemic in the spring, spreading from Mecca to Egypt, and thence all along the Mediterranean seaboard. The disease almost invariably appears at Mecca when the Mussulman pilgrimage takes place either in midwinter or in midsummer, and it has already broken out there. He considers the existing sanitary measures at seaports quite inadequate, and advocates a fresh organisation of quarantine and an international conference on the question. Two years hence the new railway will be completed in the Hedjaz, by which it will be possible to travel in four days from Mecca to Syria, and this will considerably facilitate the spread of the disease. In fact, according to the Professor, two years hence al' Europe will be at the mercy of a cholera outbreak at Mecca! A further source of danger is suggested in the emigration to America. Thousands of emigrants from Southern and Eastern Europe leave for America by French ports, chiefly Havre and Cherbourg, and the disease may easily be carried westward in this manner. The prediction is discomforting, and its realisation is certainly not improbable unless adequate measures of prevention are taken.

It is hardly necessary for us to refer to the fact that the new Act providing for the compulsory notification of births has given rise to much dissatisfaction in the profession, and a great deal of discussion in all parts of the country. In some parts the adoption of the Act has been postponed pending a possible modification of the Act in the immediate future, while in others it has been adopted in spite of protests on the part of the profession in the locality. In Bradford a satisfactory *modus operandi* appears to have been arrived at, and we commend it to the attention of the authorities and of medical men elsewhere. The medical officer of health has agreed to supply medical practitioners with letter cards to the following effect: A —— child was born at —— at ——o'clock on ——, Dr. —— being in attendance. The doctor in attendance is instructed simply to give this card to the father of the child, or any other person present, the father or other person filling up the details. In this way the M.O.H. will have done his duty in sending the cards to the medical practitioners, and they again are relieved of the undesirable duty of notifying the birth to the authorities, having reason to believe that the duty has been executed by some other person. This method has been evolved after considerable negotiation between the medical officer of health and Dr. Metcalfe, the hon. secretary of the local division of the British Medical Association. It complies with the provisions of the Act, and at the same time frees the practitioner from any obnoxious obligations. It may not ensure the notification of every birth in the district, but for that the imperfections in the Act will alone be responsible.

It would be interesting to know how far the American tinned meat scandals have permanently influenced the consumption of this class of food by the public. Such disclosures make much stir at the time, and give rise to much indignation, but they are soon forgotten, and a little assurance on the part of the retailers soon wins back the confidence of their customers in an article of food that is both palatable and convenient to the housekeeper. There is no doubt, however, that the traffic in tinned meats is still a long way from being free from suspicion, as shown by the results of recent inspection and investigation on the part of the Manchester Port Sanitary Authority. During the last five weeks of 1907 233 vessels were inspected, and of these 92 were found to be insanitary, while the inspection of food resulted in the condemnation of over 14 tons of wheat, corn, canned meats and fruits, as well as $1\frac{1}{2}$ tons of meat. It was ascertained that large quantities of old American canned meats are brought to the port of Manchester by coasting vessels after having been stored for a considerable time in this country. But it was also found that a large amount of this class of goods was coming direct from America. Out of a consignment of 1,200 six-pound tins of meat direct from New York 157 tins were blown or burst, or otherwise imperfect, and 158 were doubtful. The labels showed signs of being very old, and most of the condemned tins bore the date 1901. In order to safeguard the public it is most essential that this traffic should receive thorough inspection and supervision, and it would be well if other port authorities of the United Kingdom were equally alive to this duty.

